# Supplementary material for: Evaluation of the Xpert MTB/XDR test for detection of isoniazid, fluoroquinolones, and second-line injectable drugs resistance to Mycobacterium tuberculosis—Anhui Province, China
Source: PLoS One. 2025 Sep 12;20(9):e0331264. doi: 10.1371/journal.pone.0331264 (PMC12431546; doi:10.1371/journal.pone.0331264)
Supplement: S1 Table — (DOCX) [file pone.0331264.s001.docx]

**Supporting information**

**S1 Table. Primers of Sanger sequencing for detecting INH and FLQ resistance sites.**

| Drug | Resistance locus | Primer | Product size | Reference |
| --- | --- | --- | --- | --- |
| INH | KatG | Forward- GGCGATGAGCGTTACAGC | 670 | [10] |
|  |  | Reverse- CCAAGGTATCTCGCAACGG |  |  |
|  | fabG1-inhA | Forward- CCTCGCTGCCCAGAAAGGGA | 248 | [10] |
|  |  | Reverse- ATCCCCCGGTTTCCTCCGGT |  |  |
|  | oxyR-ahpC | Forward- GACCGGCTTCCGACCA | 472 | [10] |
|  |  | Reverse- AACTCGTCATTGAGCTTGCTG |  |  |
| FLQ | gyrA | Forward- TCGACTATGCGATGAGCGTG | 415 | [11] |
|  |  | Reverse- GGTAGCACCGTCGGCTCTTG |  |  |
|  | gyrB | Forward- CCGCTGTGATCTCGGTGAAG | 775 | [11] |
|  |  | Reverse- AGACCCTTGTACCGCTGAATG |  |  |
